# Genome-Wide Genetic Diversity and Population Structure of *Sillago sinica* (Perciformes, Sillaginidae) from the Coastal Waters of China: Implications for Phylogeographic Pattern and Fishery Management

**DOI:** 10.3390/biology14101329

**Published:** 2025-09-26

**Authors:** Tianyan Yang, Yan Sun, Peiyi Xiao

**Affiliations:** Fishery College, Zhejiang Ocean University, Zhoushan 316022, China; sunyan23@zjou.edu.cn (Y.S.); xiaopeiyi@zjou.edu.cn (P.X.)

**Keywords:** *Sillago sinica*, whole-genome resequencing, single nucleotide polymorphism, population genetics, phylogeographic pattern, fishery management

## Abstract

The ability to detect population structure and determine the extent of genetic variation among populations is critical for understanding genetic background and effective fishery management. Fifty-eight individuals of *S. sinica* were resequenced with an average depth of 24× based on the Illumina sequencing platform. A total of 7,409,691 high-quality single nucleotide polymorphisms (SNPs) and 327,698 linkage disequilibrium-pruned SNPs were detected by comparing with the reference genome, and the average nucleotide diversity (*π*) and polymorphism information content (*PIC*) for all SNPs were 0.0036 ± 0.0023 and 0.2358 ± 0.1013, respectively, indicating the relatively low level of genetic diversity caused by limited gene flow and small effective population size (*N*_e_). Integrated analyses of principal component analysis (PCA), ADMIXTURE, fixation index (*F*_st_), and cladogram showed a significant genetic divergence between the north group (Dongying and Rushan populations) and the south group (Wenzhou and Zhoushan populations), which might be related to the differences in natural and geographical environments. The comprehensive results confirmed the genetic heterogeneity of *S. sinica* populations from the northern and southern sea areas of China, and suggested that regionalization fishery management should be adopted for further resource protection and utilization of *S. sinica*.

## Simple Summary

*Sillago sinica* is a recently described Sillaginidae species along the coasts of China. Its population genetic characteristics remain largely unknown. In this study, we used whole-genome resequencing (WGR) to analyze the fine-scale genetic diversity and spatial population structure of *S. sinica* from four geographic locations: Dongying, Rushan, Wenzhou, and Zhoushan. Results revealed a relatively low level of genetic diversity and clear genetic differentiation among different populations. The findings highlight the need for region-specific fishery management to better conserve and utilize *S. sinica* resources.

## 1. Introduction

Sillaginidae fishes, commonly known as smelt-whitings, are the commercial and sport fishing targets of the inshore fisheries in the Indo-Pacific countries [1,2]. In China, they are collectively nicknamed “sand drill” due to their unique behavior associated with shallow-burying themselves under the sand when faced with a disturbance or threat. *Sillago sinica*, a new member of family Sillaginidae, was first identified and recorded from the estuaries of the Feiyun River in Wenzhou and the Yellow River in Dongying, respectively [3]. Subsequently, Korean scholars reidentified *S. parvisquamis* collected from Gwangyang and confirmed it was actually *S. sinica* according to morphological and molecular data [4]. The body color of *S. sinica* is yellowish-brown dorsally and silver ventrally, with three to four rows of dusky spots on the second dorsal fin membrane. As a short-distance migratory fish, *S. sinica* has relatively fixed spawning grounds, and only inhabits the sandy or silty substrates of the continental shelves from the Bohai Sea to the northern South China Sea [5]. *S. sinica* typically occupies mid-to-lower trophic levels in coastal and estuarine ecosystems. The stability of its population is essential for maintaining the structural and functional integrity of the food web, thereby playing a key role in sustaining overall ecosystem balance. Additionally, this fish species also possesses an important economic value and contributes significantly to supporting coastal fisheries. Nowadays, related studies of this fish are mainly concerned about fishery ecology, reproductive biology, and population genetics [6,7,8,9,10].

The northwestern Pacific is characterized by unique tectonic and hydrologic features with three continuous marginal seas separating eastern Asia from the Pacific Ocean [11,12]. The distinctive geographical and environmental conditions provide convenience conditions for investigating the mechanisms of marine diversification and speciation [13]. Settled and semi-settled fishes are susceptible to the local environments, which more easily make them generate complicated population genetic structures. Confusingly, published studies concerning mitochondrial DNA (mtDNA) genes demonstrated a high genetic connectivity among *S. sinica* populations in the coastal waters of China [6,14], while some others involving morphometrics and microsatellite markers came to the opposite conclusions [7,15]. Nonsignificant intra-population genetic differentiation was initially found in *S. japonica*, a closely related sympatric species of *S. sinica* [16]. The subsequent genomic data provided a higher population structure resolution and revealed a genetic variation pattern relevant to the local adaptation of this fish species [17,18]. Therefore, the population genetics information for *S. sinica* is still unclear and requires in-depth analysis based on the whole-genome level.

Phylogeography, as a vigorous interdisciplinary field, has experienced a great leap-forward in development since its initial introduction by John C. Avise, an outstanding contemporary bio-geneticist [19]. From the gradual understanding of mtDNA lineages in the late 1970s to the continuous maturity of research methods in recent decades, phylogeographic perspectives have consistently challenged conventional genetic and evolutionary considerations and have built a bridge between the formerly separate disciplines of population genetics in microevolutionary and phylogenetic biology in macroevolution [20,21]. Unlike the terrestrial biota, marine fish with planktonic eggs and larvae exhibit great dispersal capability and maintain large population sizes. In addition, the highly connected habitats coupled with the weak geographic barriers in the broad ocean can often contribute to frequent migration and high-level gene flow among populations [22]. Numerous studies with neutral markers have shown weak or nonsignificant genetic differentiation of most marine fishes in the northwestern Pacific, such as *Sebastes schlegelii* [23], *Larimichthys polyactis* [24], *Nemipterus bathybius* [25], and *Acanthogobius ommaturus* [26], just to name a few. Nevertheless, with the advent and growing development of next-generation sequencing (NGS) technologies in recent decades, the intraspecific variations and phylogeographic patterns of marine fishes have gradually been cleared up on a genome-wide scale [27].

Whole-genome resequencing (WGR), a high-throughput sequencing-based genomic technique conducted across multiple samples in a population, has created an unprecedented opportunity for comprehensively characterizing the polymorphic variants in the population [28]. It has great advantages of time and cost effectiveness, dense marker coverage, high accuracy and resolution, and more comparable genomic features among species and populations [29]. Therefore, more and more WGR studies have been carried out to unravel the underlying population-level genetic heterogeneity of marine and freshwater fishes based on massive genome-wide single nucleotide polymorphisms (SNPs) and putative candidate loci subjected to divergent selection [18,30,31,32].

In recent years, a draft genome of *S. sinica* has already been published and can be accessed via a public database [33], which provides a fundamental prerequisite for population genomic studies on *S. sinica*. The existing study unveiled a geographic structure signal of three *S. sinica* populations in spite of there being no strict clusters defined by their sampling locations [9]. So, here in this study, larger sample sizes of *S. sinica* from broader geographical locations were collected along the coastal waters of China, and more detailed population genetic information was revealed through a greater abundance of high-throughput SNPs. The results will help gain a deeper understanding of population genomics differences and provide theoretical support for sustainable fishery management of *S. sinica*.

## 2. Materials and Methods

### 2.1. Sample Collection and Genomic DNA Extraction

**Figure 1:**
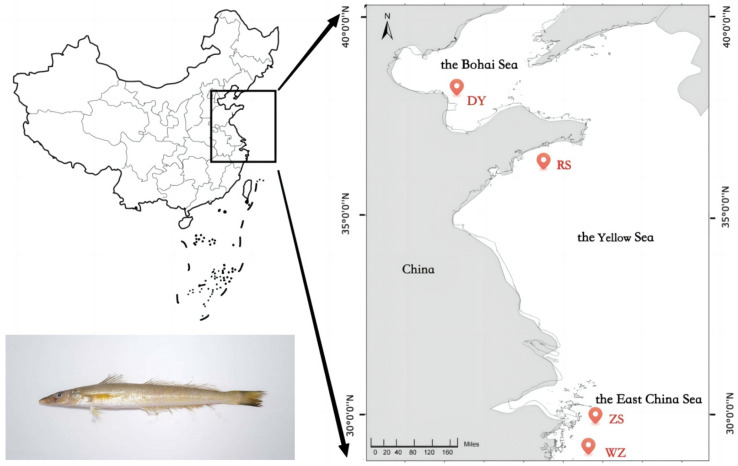
The sampling locations of *S. sinica*.

In this study, a total of 58 *S. sinica* samples from four locations were collected during September to October 2022, including Dongying (DY, *n* = 13) and Rushan (RS, *n* = 13) from the north coast of China, together with Zhoushan (ZS, *n* = 15) and Wenzhou (WZ, *n* = 17) from the south coast of China (Table 1, Figure 1). A small piece of muscle tissue was clipped from the dorsal fin base of each individual and preserved in 95% ethanol for DNA extraction. The samples were suspended in 600 μL of lysis buffer (pH = 8.0) consisting of 1 mol/L Tris-HCl, 0.5 mol/L EDTA, 10% sodium dodecyl sulfate (SDS), and 20 mg/mL proteinase K, and then incubated at 50 °C overnight. The genomic DNA was extracted using the standard phenol–chloroform method [34]. DNA extraction mainly consists of the following steps: (1) Add an equal volume of phenol and centrifuge the lysate at 12,000× *g* for 10 min at room temperature. (2) Extract the supernatant, add an equal volume of phenol/chloroform/isoamyl alcohol (25:24:1), and centrifuge the mixture. (3) Repeat extraction, add an equal volume of chloroform/isoamyl alcohol (24:1), and centrifuge again. (4) Transfer the clean aqueous phase to a new tube, add 2.5 volumes of ice-cold 100% ethanol, and store at −20 °C for two hours. (5) Centrifuge at 12,000× *g* for 15 min at 4 °C and a small white pellet which can be found at the bottom of the tube after centrifugation. (6) Add 500 mL of ice-cold 75% ethanol to wash the pellet and centrifuge again at high speed for 5 min at 4 °C. (7) Carefully remove all of the ethanol supernatant, let the pellet air-dry for 30 min, and dissolve the DNA pellet in 100 μL of TE buffer (pH 8.0). The integrity and concentration of DNA are detected by 1% agarose gel electrophoresis and Qubit 4.0 fluorometer (Thermo Fisher Scientific, Waltham, MA, USA), respectively.

### 2.2. Library Construction and High-Throughput Sequencing

The qualified template DNA was sheared into fragments of a specific size (300~500 bp) by ultrasonic mechanical interruption (Covaris M220, Woburn, MA, USA), and then the fragmented DNA was processed by purification, terminal repair, adding A-tail and sequencing adaptors, and fragment size selection using agarose gel electrophoresis. Subsequently, the appropriate DNA fragments were amplified by PCR to construct the paired-end libraries (PE150). All procedures for library construction followed the manufacturer’s protocol [35]. The prepared library was sequenced on an Illumina HiSeq 2500 (Illumina, San Diego, CA, USA) based on sequencing by synthesis (SBS) technology.

### 2.3. Sequencing Data Filtering and Reads Mapping

The original sequencing data were purified by removing adaptors, primers, and low-quality reads with quality scores lower than 20 (Q < 20) by using Cutadapt v4.4 with default parameters [36]. The clean reads were aligned to the *S. sinica* reference genome (http://gigadb.org/dataset/100490, accessed on 15 May 2024) by BWA v0.7.17 [37]. The MarkDuplicates function of the Picard tool (https://broadinstitute.github.io/picard/, accessed on 15 May 2024) was used to eliminate PCR duplicates from the filtered sam files. Alignment files were converted to bam format and then sorted according to reference sequence and coordinates using SAMtools v1.4 [38].

### 2.4. SNP Calling and Annotation

Variants were called using the HaplotypeCaller algorithm of GATK v4.0 [39]. Each sample generated its own gVCF file. These files were merged using the CombineGVCFs module, then joint genotyping analysis was performed using the GenotypeGVCFs module, and finally VCF files with all samples genotyped were obtained. The VariantFiltration module was applied for SNP filtering with the parameters “QualByDepth < 2.0, FisherStrand > 60.0, RMSMappingQuality < 40.0, MQRankSum < −12.5 and ReadPosRankSum < −8.0”. PLINK v2.0 was performed to prune the filtered SNP dataset with the option “--indep-pairwise 50 10 0.1” [40]. SNP annotation was carried out according to the genome by ANNOVAR (https://annovar.openbioinformatics.org/en/latest/, accessed on 27 June 2024) [41].

### 2.5. Genetic Diversity, Population Structure, and Genetic Differentiation Analysis

VCFtools v4.2 was used to estimate the nucleotide diversity (*π*), observed heterozygosity (*H*_o_), polymorphism information content (*PIC*), and population fixation statistics (*F*_st_), respectively [42]. A distance matrix was calculated using FastTree v1.6.0, and the phylogenetic tree was constructed using the maximum parsimony (MP) method, a classical and simple algorithm that calculated the minimum number of evolutionary steps, including nucleotide insertions, deletions, or substitutions, between species [43].

Principal component analysis (PCA) was conducted to infer the population structure of *S. sinica* based on the individual’s genomic SNP differences. It was performed using the PLINK v2.0 and the first three eigenvectors were plotted to display the individual clusters [40]. ADMIXTURE v1.3.0 software with the block relaxation algorithm was implemented to compute the ancestry proportions (Admixture) with K values preset from 1 to 10 [44]. The Monte-Carlo Cross-Validation (MCCV) was performed to derive the optimal number of clusters (K) according to the error rates, and the K value exhibiting the lowest cross-validation error (valley value) was the best one.

In order to check the effects of geographic distance and environmental differences on genetic structure, the isolation-by-distance (IBD) was assessed through the Mantel test in Distance Web Service (IBDWS) [45], and isolation-by-environment (IBE) was computed using Euclidean distances with the R package v4.3.1. The geodesic geographic distance among each pair of populations was obtained from Google Maps, and the annual mean water temperatures (30 m depth, during the years 2015 to 2022) were extracted from National Centers for Environmental Information (NCEI) (https://www.ncei.noaa.gov/access/world-ocean-atlas-2023f/, accessed on 3 July 2024), respectively.

### 2.6. Linkage Disequilibrium and Effective Population Size

Linkage disequilibrium (LD) is a statistical dependency of the DNA content that refers to a non-random correlation of neighboring alleles at nearby locations within a population [46]. In order to evaluate LD decay among different populations of *S. sinica*, we calculated pairwise correlation coefficient (*r*^2^) values within 300 kb by using PopLDdecay v3.41 software [47], and the LD attenuation diagram was visualized by R package v4.3.1.

Effective population size (*N*_e_) is an important parameter in population genetics and is related to the history dynamics of population size changing over time, which helps to evaluate population evolutionary history and conservation status [48]. The SMC++ v1.15 program, a new statistical tool capable of analyzing orders of magnitude more samples requiring only unphased data, was employed to infer the *N*_e_ and split times with a minor allele frequency (MAF) > 0.05 to effectively reduce the risk of false-negative results [49].

## 3. Results

### 3.1. Sequencing Data Statistics and Alignment

High-throughput sequencing of 58 samples generated a total of 819.4 Gb of clean data after quality control. The clean reads of each sample ranged from 64,761,454 to 163,582,108, with a total of 5,568,840,848. The average mapping ratios to the reference genome and assembly coverage were 98.33% and 98.74%, respectively. The coverage depth per individual was from 18.42× to 36.72×, averaging at 23.96× (Table 2). The quality-controlled data could meet the standards for subsequent whole-genome variation detection.

### 3.2. Screening and Annotation of SNPs

**Figure 2:**
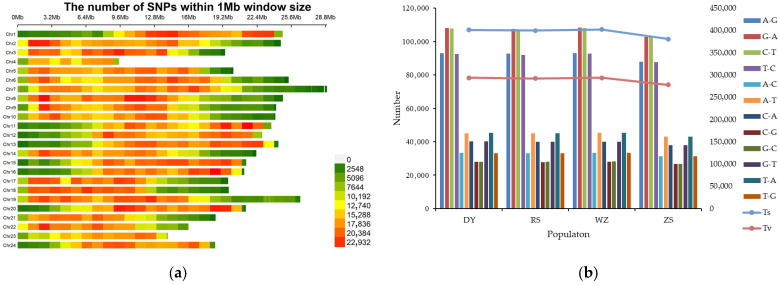
The distribution and composition characteristics of SNPs in the whole genome. (**a**) SNP density distribution; (**b**) SNP type distribution.

The distribution of genome-wide SNPs is shown in Figure 2a, and these SNPs mostly concentrated in the intercalary regions of the chromosomes. The average value of transitions/transversions (Ts/Tv) was 1.369. The main types of single base mutation were G/A and C/T, followed by A/G and T/C (Figure 2b). A total of 7,409,691 high-quality SNPs and 327,698 LD-pruning SNPs in 58 samples were identified after screening. Based on the genome annotation, SNPs were categorized into exonic regions, intronic regions, splicing sites, 3′UTRs and 5′UTRs, upstream and downstream regions, and intergenic regions. In this study, most of the SNPs were located in the intronic and intergenic regions, accounting for 46.71% and 38.46%, respectively. About 324,630 loci were located in coding exons, and these SNPs could be further grouped into synonymous SNPs (216,335) and nonsynonymous SNPs (106,666). Moreover, mutations causing stop gain and stop loss were also classified, with numbers of 1443 and 186, respectively (Table 3).

### 3.3. Genetic Diversity and Differentiation

**Figure 3:**
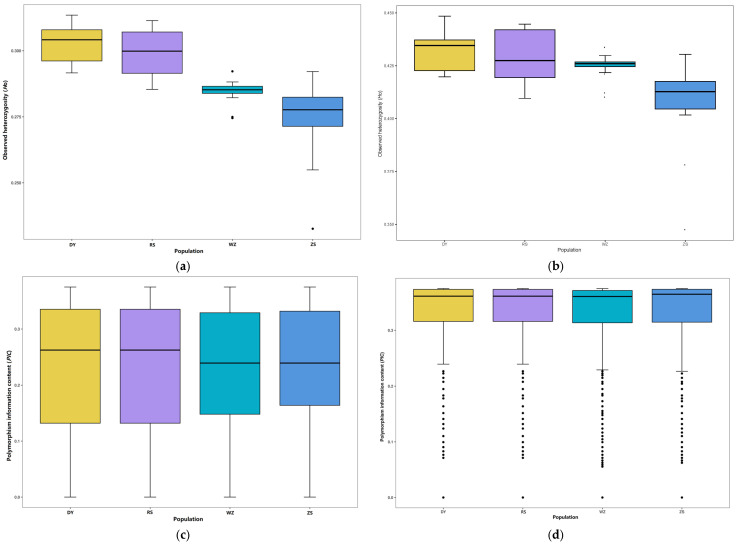
Population genetic parameters of *S. sinica.* (**a**) Observed heterozygosity (*H*_o_) for all SNPs; (**b**) observed heterozygosity (*H*_o_) for LD-pruning SNPs; (**c**) polymorphism information content (*PIC*) for all SNPs; (**d**) polymorphism information content (*PIC*) for LD-pruning SNPs.

The values of average nucleotide diversity (*π*) for all SNPs and LD-pruning SNPs were, separately, 0.0036 ± 0.0023 and 0.0002 ± 0.0002 across all populations. The actual number of heterozygotes, also called observed heterozygosities, for all SNPs were 0.3031 ± 0.0075 (DY), 0.2986 ± 0.0089 (RS), 0.2745 ± 0.0146 (ZS), and 0.2847 ± 0.0043 (WZ), respectively (Figure 3a). After LD pruning, the observed heterozygosities were 0.4330 ± 0.0098 (DY), 0.4291 ± 0.0120 (RS), 0.4077 ± 0.0207 (ZS), and 0.4246 ± 0.0059 (WZ), respectively (Figure 3b). Polymorphism information content (*PIC*) is a basic characteristic of SNP marker informativeness that is often used for measuring the degree of polymorphism. The *PIC* values based on all SNPs and LD-pruning SNPs varied from 0.2320 ± 0.1010 to 0.2402 ± 0.1017 and from 0.3183 ± 0.0864 to 0.3214 ± 0.0849, with an average of 0.2358 ± 0.1013 and 0.3199 ± 0.0858, respectively (Figure 3c,d).

**Figure 4:**
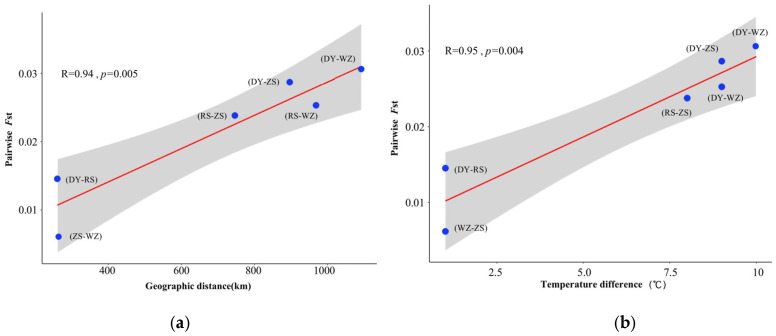
The correlations among genetic, geographic, and environmental distances. (**a**) Isolation-by-distance (IBD) test; (**b**) isolation-by-environment (IBE) test. The red line represents regression line, and the gray area represents the 95% confidence interval. The notation DY-RS represents Dongying population versus Rushan population, and so forth.

The pairwise *F*_st_ values between populations based on all SNPs were calculated. The highest *F*_st_ value was 0.0306 ± 0.0293 (*p* < 0.05) between DY and WZ populations, while WZ and ZS populations had the weakest genetic differentiation with the lowest *F*_st_ value of 0.0061 ± 0.0124 (*p* > 0.05) (Table 4). The pairwise *F*_st_ values obtained from LD-pruning SNPs had the same trend. The genetic divergence pattern among populations of *S. sinica* could be explained by the IBD test (R = 0.94, *p* = 0.005) and IBE test (R = 0.95, *p* = 0.04), which showed that genetic differentiation increased with geographic distance or environmental differences between populations (Figure 4).

### 3.4. The Hierarchical Genetic Structure and Phylogenetic Relationships

**Figure 5:**
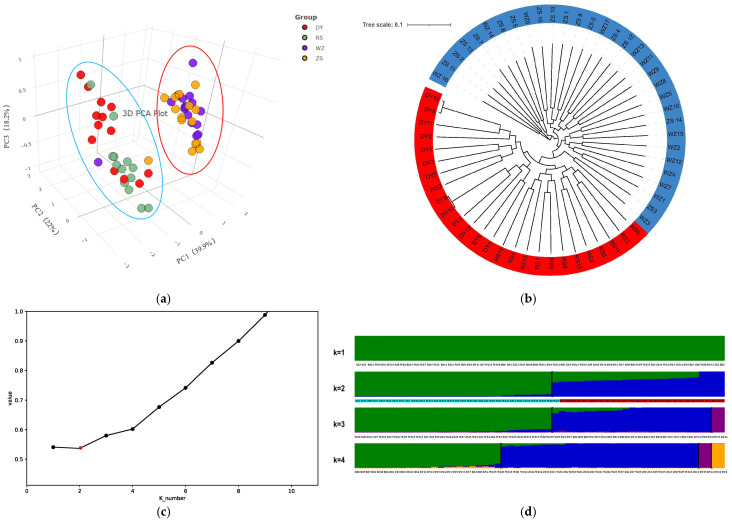
Analysis of the population structure in *S. sinica*. (**a**) The PCA plot of the first three PCs; (**b**) the phylogenetic tree; (**c**) cross-validation error rate for each K value in Admixture. The red dot represents the lowest cross-validation error; (**d**) analysis of Admixture with the assumed number (K = 1 to 4). Each column represents an individual, where different colors represent different ancestors and the length of different colored segments represents the proportion of an ancestor in the individual’s genome.

For each principal component (PC), the proportions of variances were 0.399 (PC1), 0.220 (PC2), and 0.182 (PC3), suggesting that the corresponding components held much interpretive value and captured the variability in the data [50]. Thus, the first three PCs were extracted and plotted by PCA dimensionality reduction. The 3-dimension scatter plot indicated that DY and RS populations belonging to the north group, as well as WZ and ZS populations from the south group, were basically clustered together, respectively (Figure 5a). Meanwhile, the result of the evolutionary tree also revealed that all individuals of *S. sinica* were differentiated into two branches. One branch contained DY and RS populations, and the other one included ZS and WZ populations (Figure 5b).

The potential population genetic structure of *S. sinica* was further inferred by Admixture analysis, with the cluster numbers (K) initially set from 1 and 1 added for each repetition for all the 58 individuals. The lowest cross-validation error value was obtained in the case of K equal to 2 (Figure 5c), demonstrating that it was the most likely number of genetic clusters. When K = 1, all individuals were well-mixed. In the model with K = 2, four populations were generally separated into two groups; one group was composed of WZ and ZS populations, and the other group was formed by DY and RS populations (Figure 5d). Each group identified by population structure analysis had its unique hereditary constitution, even if the groups shared the same ancestry. The above results showed a consistent clustering trend with PCA and phylogenetic analysis.

### 3.5. Demographic History Inference

**Figure 6:**
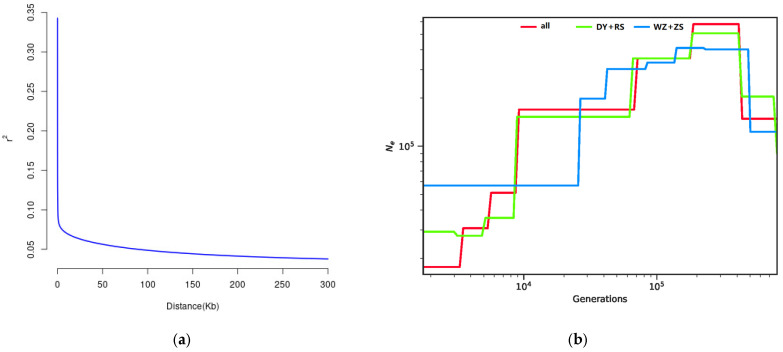
The demographic history of *S. sinica* populations. (**a**) The linkage disequilibrium analysis; (**b**) the SMC++ model assesses effective population size changes. DY+RS represents Dongying and Rushan populations, while WZ+ZS represents Wenzhou and Zhoushan populations.

LD patterns across the genome could be affected by evolutionary forces including mutation, genetic drift, natural selection, and recombination [51]. Hence, LD map is a useful tool to study genetic diversity [52]. The extent of LD for high-quality SNPs was evaluated and the average *r*^2^ values decreased by increasing the marker distance between pairwise SNPs, reflecting a rapid descent over the first 1 kb (Figure 6a). Using above-mentioned information, we estimated the historical population dynamics over the past 106 generations (generation time = 1, mutation rate = 3.6 × 10^−9^) when the common ancestor of *S. sinica* had not yet diverged (Figure 6b). The estimation from the SNP datasets showed the north group, as well as all individuals, demonstrated an initial decline in population size over the past 100,000 years, and then a burst with the greatest decrease occurring in the last ~10,000 years, suggesting that *S. sinica* from the coasts of China experienced genetic bottleneck and the genetic diversities remained at a low level. A similar tendency was found between all 58 individuals and the north group. However, the north and south groups experienced slightly different demographic trajectories, with the south group (WZ and ZS populations) suffering the bottleneck effect earlier than the north group (DY and RS populations) and showing a drastic decay at about 25,000 years before present.

## 4. Discussion

### 4.1. Genetic Diversity and Variation

Genetic diversity is a foundation for species diversity and ecosystem diversity, which commonly reflects the genetic variations among different populations within a species [53]. In a sense, genetic diversity has become a prerequisite for the adaptive evolution of species. The genetic diversity level of *S. sinica* along China’s coasts has only ever been evaluated by some other mtDNA markers, such as control region (*π* = 0.001073~0.005399) and Cyt *b* gene (*π* = 0.0000~0.0043) [6]. Herein, we used genome-scale SNPs to reveal the genetic variation in *S. sinica*. Compared with some marine fishes with higher genetic diversities detected by genomic sequencing technologies, such as *Pampus minor* (*π* = 0.15303~0.15688) [54], *Harpadon nehereus* (*π* = 0.63664~0.74868) [55], and *Konosirus punctatus* (*π* = 0.18~0.26) [56], a somewhat lower genetic diversity was observed in *S. sinica*, which was coincident with previous findings of mtDNA, nuclear DNA (nDNA), and SNP markers [6,7,9]. This diminished genetic diversity of *S. sinica* likely resulted from limited gene flow without long-distance migration just like some other estuarine fishes [18,57,58].

Genetic diversity decreases with a reduction in effective population size and an increase in the effects of genetic drift [59]. In the presumptive pattern of historical demography, the continuous reduction in effective population size after undergoing a population bottleneck might quickly reduce the population genetic diversity of *S. sinica*. Genetic drift in small populations usually acts more quickly to diminish genetic variation. It was confirmed that scarce species have almost always had lower levels of genetic diversity than high-density ones [60]. As a newly discovered and less common species in China, *S. sinica* has a narrow distribution range and paucity of natural resources, which make it particularly susceptible to genetic drift.

The *H*_o_ and *PIC* are the other two main indices used for assessing the population genetic diversity. The data presented in the boxplots implied that the DY and RS populations had a higher proportion of heterozygous loci than the WZ and ZS populations. The higher polymorphism level of the DY and RS populations hinted that they might possess more complex population structures and stronger environmental adaptability, while the stochastic loss of rare alleles caused by the environmental changes and overfishing presumably led to a decrease in genetic diversity in WZ and ZS populations [61].

### 4.2. Genetic Differentiation Caused by Historical and Contemporary Population Dynamics

In the presently reported study, a comparative genomics approach was used to define the population structure of *S. sinica* along China’s coasts. In contrast to the conclusions obtained by mtDNA markers [6,14], a significant genetic differentiation among the populations of *S. sinica* was also detected by comprehensive population genetic analyses of PCA, ADMIXTURE, and MP tree, implying that *S. sinica* along China’s coasts could be divided into two genetic clusters. The DY and RS populations form a group located on the north coast of China, and the WZ and ZS populations form another separate one. Our results revealed that the phylogeographic pattern of *S. sinica* in the northwestern Pacific could be classified as “major allopatric lineages with weak to moderate differentiation”, defined by Avise [20]. The north and south populations probably became separated by the Yangtze River estuary, and this long-term geographical barrier hindered genetic communication and caused the two isolated gene pools of *S. sinica* populations. Changjiang diluted water (CDW) forms a huge water tongue with low salinity, high turbidity, and rich nutrients, which constitutes a strong physical–chemical barrier [62]. China Coastal Current (CCC), Taiwan Warm Current (TWC), and Kuroshio Current (KC) converge outside the Yangtze River estuary, and the differences in water mass properties (temperature, salinity) further strengthen the divergences of populations [63]. From the perspective of historical climate events, the last glacial–interglacial cycles led to severe fluctuations in sea level, forcing different populations into isolated refuges and creating original genetic differentiation on a time scale [64]. Similar evidence was also found in *Scylla serrata* [65], *Siphonaria japonica* [66], *Oplegnathus fasciatus* [67], and *Amphioctopus fangsiao* [68]. Furthermore, a significant genetic differentiation had occurred between DY and WZ populations, which was perhaps associated with geographic and environmental differences. More and more evidence has suggested that gene flow is not only affected by geographic distance, but also by environmental differences over the decades [69,70]. In other words, the farther the geographic distance, the greater the environmental difference and the more significant genetic differentiation. In this study, the results for IBD and IBE also confirmed that both geographic and environmental factors strongly influenced genetic differentiation of *S. sinica* populations.

Insights from genetic analyses have included evidence of postglacial colonization and population expansion. The timescale inferred by SMC++ depends on parameter assumptions. In this study, the demographic history of *S. sinica* could be traced to 100 thousand years ago (Ka), and the populations had suddenly experienced a very large decline. This phenomenon was also discovered in *S. japonica* [18]. In comparison with the present day, the sea level dropped down during the Late Pleistocene epoch of an interglacial to glacial transition stage [71]. Evidence of population expansion is often specific to particular ocean basins, and it is verified that signals of population expansion are closely linked to a period of 120~140 Ka in marginal seas of the northwestern Pacific [13]. To some extent, the estimated bottlenecks of *S. sinica* correspond to known glacial and postglacial events and reflect historical isolation between the East China Sea and the Yellow Sea–Bohai Gulf of China. Zhao et al. also validated that the subtropical population and warm-temperate populations exhibited strong genetic differentiation signals [9].

### 4.3. Fishery Resource Protection and Utilization

Knowledge of the genetic makeup of a species is crucial for effective fishery management and germplasm utilization. Nowadays, the population genomics have become a powerful tool to understand the status quo of marine biodiversity and have offered references for the sustainable development of the fishery industry [72]. A population with a small gene pool or very low genetic diversity is much less able to survive in the event of environmental changes, and so can become vulnerable to resource depletion. The genetic diversity of *S. sinica* is maintained at a low level, which reduces its adaptability to environments and elevates the risk of inbreeding depression. Consequently, once overexploited, the natural resources are unlikely to recover in a short period of time. Additionally, the significant genetic differentiation between the north and south populations of *S. sinica* manifests such that the impacts of environmental and geographical factors must be taken into consideration. Hence, fishery managers and relevant departments must establish a biogeography-based management framework for *S. sinica* that includes integrated ecological and long-term monitoring.

A stock enhancement program is an essential means to restore resources and maintain genetic diversity [73]. At present, researchers have successfully overcome the technical difficulties of artificial reproduction in *S. sihama*, and it has become a target fish species for marine aquaculture in China [74,75]. The experiences of artificial propagation and stocking of *S. sihama* should be leveraged. Tentative propagation and stock enhancement projects can be implemented to increase the wild populations of *S. sinica*, so as to achieve the goal of reasonable protection and sustainable utilization.

## 5. Conclusions

In the present study, we employed an Illumina sequencing platform to characterize the genome-scale genetic variation, phylogeographic patterns, and historical population dynamics of *S. sinica* across four sampling locations in coastal waters of China. The populations of *S. sinica* were characterized by relatively lower levels of nucleotide diversity and heterozygosity. Analysis of population structure revealed regional genetic differentiation between the northern and southern populations, consistent with a pattern of isolation by geographical and historical barriers to gene flow. The demographic reconstruction showed evidence of historical population declines during the Quaternary glacial epoch. The conclusions serve as a reference for fishery resource management of *S. sinica*.

The abundance of variable sites from genome resequencing facilitates genome-wide scans for natural selection. In the near future, the selective signals associated with growth, metabolism, and the immune and environmental tolerance of *S. sinica* will be detected to elucidate the genetic mechanism of adaptive evolution.

## Figures and Tables

**Table 1:** Sample information of *S. sinica* in this study.

Population	Sampling Date	Number	Body Length (cm)	Body Weight (g)
DY	16 September 2022	13	13.40–18.20	17.88–39.59
RS	7 October 2022	13	12.25–16.61	14.67–47.59
WZ	5 September 2022	17	15.62–20.30	20.45–67.38
ZS	20 September 2022	15	16.40–20.70	24.95–73.05

Abbreviations: DY, RS, WZ, and ZS mean Dongying, Rushan, Wenzhou, and Zhoushan, respectively.

**Table 2:** The statistics sequencing results of *S. sinica*.

Population	Number	Average Raw Reads	Average Clean Reads	Average Mapping Rate (%)	Average Coverage (%)	Average Sequencing Depth (×)	Q20 (%)	Q30 (%)
DY	13	83,880,884	81,054,146	99.27	98.80	22.11	96.30	90.84
RS	13	88,775,733	85,728,715	99.34	98.84	23.37	96.18	90.59
WZ	17	92,952,993	90,221,569	98.74	98.81	24.25	95.90	89.82
ZS	15	124,514,599	124,459,799	96.16	98.51	25.69	93.29	82.83
Total	58	98,145,791	96,014,497	98.33	98.74	23.96	95.38	88.41

Q20 and Q30 mean 1% and 0.1% base-call errors in a sequencing dataset, respectively.

**Table 3:** Functional annotation of identified SNPs.

Category		Number	Percentage (%)
Total		7,409,691	100.00
Upstream (1 kp)		285,912	3.86
Downstream (1 kb)		246,947	3.33
Upstream/downstream (1 kb)		47,169	0.64
3′UTRs		145,709	1.97
5′UTRs		46,762	0.63
Splicing		1372	0.02
Intergenic		2,849,683	38.46
Intronic		3,461,507	46.71
Exonic		324,630	4.38
	Nonsynonymous SNV	106,666	32.86
	Synonymous SNV	216,335	66.64
	Stop gain	1443	0.44
	Stop loss	186	0.06

UTRs mean untranslated regions and SNV means single nucleotide variant.

**Table 4:** The *F*_st_ values between different *S. sinica* populations.

	DY	RS	WZ	ZS
DY				
RS	0.0145 ± 0.0215 (0.0145 ± 0.0228)			
WZ	0.0306 ± 0.0293 * (0.0291 ± 0.0307) *	0.0253 ± 0.0263 (0.0256 ± 0.0302)		
ZS	0.0287 ± 0.0275 (0.0287 ± 0.0310)	0.0238 ± 0.0256 (0.0246 ± 0.0295)	0.0061 ± 0.0124 (0.0077 ± 0.0164)	

Abbreviations DY, RS, WZ, and ZS mean Dongying, Rushan, Wenzhou, and Zhoushan, respectively. Numbers in brackets denote the *F*_st_ values based on LD-pruning SNPs, and the asterisks represent significant differences (*p* < 0.05).

## Data Availability

All the sequencing data have been deposited in GenBank (https://www.ncbi.nlm.nih.gov/, accessed on 11 April 2025) under the bioproject accession number PRJNA1248230.
